# Genomic Predictors of Diapause Polymorphism in the Bark Beetle *Ips typographus* With Implications for Voltinism Potential and Outbreak Dynamics

**DOI:** 10.1111/mec.70532

**Published:** 2026-08-25

**Authors:** Luciano Palmieri, Edwina J. Dowle, Krystyna Nadachowska‐Brzyska, Axel Schopf, Nina Dobart, Christian Stauffer, Hannes Schuler, Gregory J. Ragland, Martin Schebeck

**Affiliations:** ^1^ Faculty of Agricultural, Environmental and Food Science Free University of Bozen‐Bolzano Bolzano Italy; ^2^ Competence Centre for Plant Health Free University of Bozen‐Bolzano Bolzano Italy; ^3^ Department of Integrative Biology University of Colorado–Denver Denver Colorado USA; ^4^ New Zealand Institute for Bioeconomy Science Limited Lincoln New Zealand; ^5^ Institute of Environmental Sciences, Faculty of Biology Jagiellonian University Kraków Poland; ^6^ Institute of Forest Entomology, Forest Pathology and Forest Protection, Department of Ecosystem Management, Climate and Biodiversity BOKU University Vienna Austria; ^7^ Department of Forest Entomology, Faculty of Forest Sciences and Forest Ecology University of Göttingen Göttingen Germany

**Keywords:** dormancy, juvenile hormone esterase, machine‐learning, phenology, seasonality

## Abstract

Diapause polymorphisms shape insect life cycles, affecting their responses to seasonal environmental fluctuations. In the spruce bark beetle *Ips typographus*, diapause phenotypes, classified as facultative or obligate, significantly impact voltinism and thus outbreak potential, yet the genetic mechanisms underlying this trait remain incompletely understood. Here, we combined reduced‐representation genome‐wide association study (rGWAS) and machine learning (ML) to identify genetic polymorphisms associated with diapause phenotypes. Using ddRADseq, we analysed genomic data from diapause‐phenotyped individuals originating from distinct geographic locations in Central and Northern Europe. rGWAS identified SNPs associated with diapause phenotypes, with a locus encoding juvenile hormone esterase (JHE) emerging as the strongest and only association robust to empirical null calibration. ML classification independently recovered JHE, detected additional loci involved in protein regulation, signal transduction and stress‐response pathways, and, following permutation and cross‐population validation, achieved high predictive accuracy. Our combined rGWAS and ML approach outperformed either method alone, effectively identifying a minimal set of SNPs capable of robustly distinguishing facultative from obligate diapause phenotypes. Applying our model to wild, non‐phenotyped *I. typographus* populations revealed a high proportion (> 50%) of facultative diapause phenotypes (potentially multivoltine) in Central European regions and obligate diapausing individuals (~97%; univoltine) prevailing at northern latitudes, consistent with adaptive responses to local environmental conditions and demonstrating the feasibility of inferring voltinism potential from genomic data in natural populations. Our study provides significant advances for ecological genomics research and presents new tools for predicting and managing outbreak risks in pest species under climate change.

## 1 Introduction

Insects encountering seasonally fluctuating environments require adaptive strategies to synchronize their life cycles with periods favourable for growth and reproduction, while mitigating the impacts of stressful conditions. Dormancy is an evolutionary adaptation that insects employ to respond to these temporal challenges (Denlinger 2022; Koštál 2006). Dormancy responses are diverse but can generally be sorted into one of two broadly defined physiological states: quiescence, an immediate response directly triggered by environmental conditions, and diapause, a hormonally regulated and genetically programmed alternative developmental pathway, usually entered preemptively when environmental conditions are still favourable (Koštál 2006; Schebeck et al. 2024). Diapause is characterized by suppressed development or markedly slowed developmental processes, reduced metabolic activity, reproductive arrest and enhanced tolerance to environmental stressors (Denlinger 2022; Hahn and Denlinger 2011; Schebeck et al. 2024).

Diapause is a dynamic process comprising distinct phases, including induction, preparation, initiation, maintenance and termination, and is either facultative, that is, entered in response to environmental signals, or obligate, that is, expressed as part of an insect's life cycle, irrespective of environmental factors (Koštál 2006; Ragland et al. 2019). Facultative diapause is commonly induced by photoperiod or temperature, which signal upcoming seasonal changes (Denlinger 2022; Koštál 2006). The timing of diapause induction has considerable implications for insect seasonal timing (phenology), affecting activity, reproduction and voltinism (the number of generations per year), and consequently population dynamics and adaptations to local environments (Numata and Shintani 2025; Schebeck et al. 2024). Facultative diapause allows species to flexibly adjust their voltinism to prevailing conditions, whereas obligate diapause constrains insects to univoltine (or even semivoltine) life cycles (Koštál 2006; Numata and Shintani 2025).

The physiological nature and timing of diapause critically determine ecological characteristics, such as survival and geographic distributions, and evolutionary outcomes, such as rapid adaptation to changing climates (Bradshaw and Holzapfel 2001; Gomi and Takeda 1996) and ecological speciation (Numata and Shintani 2025). Understanding the genomic underpinnings of diapause traits has broad implications for comprehending adaptive responses to environmental challenges across insect taxa. At the molecular level, progression through diapause phases involves complex changes in the expression/abundance of many genes and metabolites (Lehmann et al. 2018). However, the genetic architecture underlying variation in various metrics of diapause can range from one or a few major effect loci to highly polygenic (Koštál et al. 2017; Pruisscher et al. 2022). Transcriptional profiling and genomic studies in various insects revealed that although certain physiological mechanisms and hormonal pathways are conserved, the specific genetic basis of diapause can vary substantially among taxa, emphasizing the need for species‐specific investigations (Dowle et al. 2020; Koštál et al. 2017; Ragland and Keep 2017). Diapause‐associated genes and pathways commonly found across insects include those governing hormonal regulation, particularly juvenile hormone (JH) and ecdysteroids, and associated enzymes like juvenile hormone esterase (JHE), as well as core signalling cascades such as the insulin, TOR and Wnt pathways (Koštál et al. 2017; Ragland et al. 2010, 2011; Sim and Denlinger 2013). Furthermore, genes involved in metabolic processes, energy storage, stress response, cell cycle control and photoperiodic perception are often key components of diapause regulation (Hahn and Denlinger 2011; Koštál 2006; Koštál et al. 2017; Ragland et al. 2010, 2011; Sim and Denlinger 2013). Additionally, specific genes related to reproductive development, such as those involved in oogenesis, are also frequently linked to the expression of reproductive diapause (Sim and Denlinger 2013).

The spruce bark beetle *Ips typographus* (L.), represents an ideal model for examining the adaptive significance of diapause polymorphism due to its pronounced ecological impacts and its geographically variable facultative and obligate diapause strategies (Doležal and Sehnal 2007; Schebeck et al. 2022). *Ips typographus* is recognized as the most destructive insect pest of Norway spruce, *Picea abies* , the dominant tree species in forests across Eurasia. During eruptive population outbreaks, *I. typographus* can cause substantial tree mortality, triggering major ecological disturbances and economic losses (Hlásny et al. 2021). Climate change has exacerbated bark beetle impacts by increasing the frequency and severity of extreme weather events, such as storms and droughts, that compromise tree defences and render hosts more susceptible to infestation (Hlásny et al. 2021). Furthermore, elevated temperatures prolong beetle breeding periods, thereby amplifying the risk of epidemic outbreaks (Hofmann, Kautz, and Schebeck 2025; Hofmann, Schebeck, and Kautz 2025). Consequently, recent warm years have facilitated additional annual beetle generations in certain regions, increasing outbreak risks (Jaakkola et al. 2023).

A central factor influencing voltinism and thus outbreak dynamics in *I. typographus* is diapause polymorphism, which encompasses facultative and obligate diapause phenotypes (Schebeck et al. 2022). Facultative diapause is mainly regulated by short day‐lengths, but warm temperatures suppress diapause induction, allowing beetles to establish additional offspring generations late in the season (Doležal and Sehnal 2007; Hofmann, Kautz, and Schebeck 2025; Schebeck et al. 2022). Given favourable temperature conditions, facultative diapausing individuals are capable of producing multiple generations per year. In contrast, individuals entering obligate diapause have a univoltine life cycle independent of environmental cues, which requires exposure to winter chilling before reproduction and growth can resume the following spring (Hofmann et al. 2024; Hofmann, Schebeck, and Kautz 2025; Schebeck et al. 2022). Populations typically harbour both diapause phenotypes in varying frequencies, facilitating flexible voltinism patterns (Schebeck et al. 2017, 2022). Surveys conducted along latitudinal and elevational gradients indicate variation in the relative abundance of diapause phenotypes, with facultative diapausing beetles dominating in southern and low‐elevation populations, whereas obligate diapausing beetles prevail in northern or high‐elevation populations where shorter growing seasons and early onset of the cold season impose developmental constraints and increased mortality risks (Schebeck et al. 2017, 2022).

Dissecting the genetic basis of complex, environmentally plastic traits such as diapause requires analytical approaches capable of detecting both individual loci with moderate‐to‐large effects and the collective contributions of many small‐effect variants. Genome‐wide association studies (GWAS) provide a hypothesis‐free framework for identifying individual SNPs statistically associated with phenotypic variation, but their power diminishes when traits are highly polygenic or when causal variants have small effect sizes (Korte and Farlow 2013). Machine learning (ML) approaches, particularly ensemble methods such as gradient boosting, complement GWAS by capturing non‐linear interactions among loci and by ranking features according to their joint predictive contribution rather than marginal association alone (Ahmed et al. 2022; Li et al. 2018). Combining GWAS and ML thus leverages the strengths of both paradigms: GWAS anchors candidate loci in a statistically rigorous association framework, while ML optimizes predictive accuracy and identifies feature combinations that may be missed by single‐marker tests.

Despite the ecological and evolutionary importance of diapause, the genetic architecture underlying diapause polymorphism in insects is not well understood. Elucidating this genetic architecture has broader implications for unravelling the genetic mechanisms underlying seasonal adaptations. To bridge this critical knowledge gap, our study leverages a high‐throughput DNA sequencing approach to infer the genetic make‐up of diapause phenotypes in *I. typographus* and to subsequently predict phenotypes from genotypes in natural populations. Specifically, our objectives were to (1) identify genomic regions linked to obligate versus facultative diapause phenotypes, (2) develop a machine learning‐based predictive model to classify individual diapause phenotypes from genomic data and (3) apply this model to infer ratios of diapause phenotypes and thus the potential voltinism of naturally occurring wild *I. typographus* populations. Our approach aims to elucidate the genetic mechanisms underlying diapause and to enhance the predictive power of bark beetle phenotyping tools for effective forest pest management under rapid environmental change.

## 2 Materials and Methods

### 2.1 Insect Collection, Diapause Phenotyping, DNA Extraction and ddRAD Sequencing

To investigate the genetic polymorphisms associated with the expression of obligate or facultative diapause phenotypes, we used *I. typographus* individuals collected from the same Central and Northern European populations as described in Schebeck et al. (2022): Prinzersdorf, Austria (48°22′ N, 15°48′ E; 350 m), Gesäuse, Austria (47°35′ N, 14°38′ E; 1500 m) and Vindeln, Sweden (64°12′ N, 19°43′ E; 300 m). For clarity, we refer to these populations hereafter as LOW (AT‐Prinzersdorf; lower altitude), HIGH (AT‐Gesäuse; higher altitude) and NORTH (SW‐Vindeln), respectively. Diapause phenotypes were determined under laboratory conditions in 2010, as described in (Schebeck et al. 2022). In short, adults were allowed to colonize fresh spruce logs under controlled temperatures and photoperiods, and diapause status of the following offspring generation was determined by assessing emergence from logs, egg maturation and ovary development to score reproductive activity (all three are reliable traits to evaluate expression of reproductive diapause in *I. typographus*; Hofmann, Schebeck, and Kautz 2025; Schebeck et al. 2022). Young adults emerging from logs under permissive photoperiods and temperatures were generally regarded as non‐diapausing, that is, facultative diapause, and beetles remaining in logs under favourable conditions were classified to express obligate diapause (Schebeck et al. 2017, 2022). Beetles that developed into reproductively active individuals under favourable temperature and photoperiodic conditions were scored as facultative diapause, whereas beetles that had immature ovaries under permissive temperatures and photoperiods were scored as obligate diapause (Schebeck et al. 2022). Phenotyped individuals were stored at −80°C until further processing. In addition, we collected *I. typographus* from the three abovementioned wild populations in 2015 (randomly sampled from naturally colonized logs; only one individual per gallery to avoid the analysis of siblings) that were not diapause‐phenotyped in the laboratory, to perform a test predicting diapause phenotypes in nature (details described below). Field‐collected beetles were immediately transferred to absolute ethanol and stored at −20°C.

DNA was extracted individually from each of the 297 *phenotyped* and 286 *wild* beetles using whole‐body tissues and the GenElute Mammalian Genomic DNA Miniprep Kit (Sigma‐Aldrich, St. Louis, MO), following the manufacturer's instructions. The *phenotyped* samples included 59 obligate and 60 facultative diapausing beetles from LOW, 61 obligate and 60 facultative diapausing beetles from HIGH, and 57 obligate diapausing beetles from NORTH. Notably, both facultative and obligate diapause phenotypes were obtained from the same geographic populations (LOW and HIGH) with approximately balanced representation, ensuring that SNP‐phenotype associations are not confounded by population origin. The NORTH population contributed only obligate diapause individuals because facultative diapause is extremely rare at northern latitudes due to environmental constraints that strongly favour univoltine life cycles (Schebeck et al. 2017, 2022). The *wild* samples included 96 beetles each from LOW and HIGH, and 94 from NORTH. DNA concentrations were measured using a ‘NanoDrop 2000C’ and samples were diluted or concentrated to ±10% of concentrations for downstream library preparations. Genome‐wide, multi‐locus genotypes were determined using a double‐digest restriction‐associated DNA (ddRAD) sequencing approach (Peterson et al. 2012). In brief, ~200 ng DNA was digested using the restriction enzymes EcoRI and MseI (New England Biolabs) and adapters were ligated to these fragments, containing unique, in‐line barcodes and forward Illumina sequencing primers (to the EcoRI end) and reverse Illumina sequencing primers (to the MseI end). These fragments were subsequently PCR‐amplified (30 cycles) using primers complementary to the adapters. Afterwards, PCR products were pooled (groups of about 96 individuals) and size selection for fragments of 300–500 bp was performed using a BluePippin system (Sage Science, Beverly, MA). Pools were then cleaned with Ampure beads (Agencourt AMPure XP, Beckman Coulter, Indianapolis, IN) and sequenced at the University of Kansas Genome Sequencing Core on an Illumina HiSeq2000 (for 100 cycles, four paired‐end, two single‐end lanes).

### 2.2 Bioinformatics

Raw reads were demultiplexed and cleaned using a custom python script as described in Dowle et al. (2017). For data analysis, only forward reads were used as the reverse reads were of low quality. After initial processing, we generated a reduced‐genome representation assembly using the ipyrad pipeline v.0.9.95 (Eaton and Overcast 2020). The assembly parameters file was configured for a reference‐based method, guided by the consensus genome from Nadachowska‐Brzyska et al. (2026), this genome assembly consists of 16 chromosomes (or linkage groups, LGs) and multiple small contigs not assembled into chromosomes. We specified the ‘*ddrad*’ flag for the data type and set the clustering threshold to 0.80; all other parameters were kept at their default settings. The resulting VCF file (containing single nucleotide polymorphisms, SNPs) was filtered with vcftools v.0.1.16 (Danecek et al. 2011) to remove loci with more than 25% missing data. High proportions of missing data are expected in ddRAD‐seq datasets due to stochastic variation in restriction site coverage across individuals (Peterson et al. 2012); we therefore removed loci with more than 25% missing data to retain only reliably genotyped sites. We then pruned the remaining loci for linkage disequilibrium using PLINK v.1.90b6.21 (Purcell et al. 2007) with the command ‘‐‐indep‐pairwise 50 10 0.2’. This LD‐pruning step collapses clusters of highly correlated variants so that each RAD tag contributes at most one representative SNP and neighbouring tags that still share allele patterns above the *r* ^2^ ~ 0.2 threshold are trimmed. The resulting panel more closely satisfies the assumption of approximate marker independence for downstream association tests, thereby avoiding inflation from pseudo‐replication. Operationally, PLINK slides a 50‐SNP window in 10‐SNP steps, computes pairwise *r* ^2^ within each window, and iteratively removes one SNP from any pair exceeding the threshold, typically the variant with lower information content. The final SNP set was split into two files, one for the *phenotyped* individuals and another for the *wild* individuals. An overview of the entire analytical workflow is presented in Figure S1. Input and configuration files, program commands utilized and scripts for all the analysis in this study are available at a public GitHub and ZENODO repositories (github.com/LucPalmieri/Diapause_genetic_markers, https://doi.org/10.5281/zenodo.17543185).

### 2.3 Population Structure and Reduced‐Representation Genome‐Wide Association Study (rGWAS)

We quantified population structure by variance‐component eigenanalysis (VCA) of the additive genomic relationship matrix (GRM; Patterson et al. 2006; Yang et al. 2011) used here as an alternative to PCA of the raw genotype covariance to account for underlying kinship among *phenotyped* individuals. First, genotype data were read from the VCF file and converted into a genlight object using *vcfR* v.1.7.0.91 (Knaus and Grünwald 2017) and *adegenet* v.1.3.1 (Jombart and Ahmed 2011).

Missing genotypes were imputed with the mean allele frequency, and a GRM was generated with *rrBLUP* v.4.6.3 (Endelman 2011). Eigenvalue decomposition of this matrix yielded variance‐component (VC) scores, the eigenvectors of the GRM, which summarize realized relatedness; mathematically, this is equivalent to principal components computed on the GRM rather than on the raw genotype covariance (Patterson et al. 2006; Price et al. 2006). All VCA analyses were performed in R v.4.4.2 (R Development Core Team 2024). We further assessed population structure with STRUCTURE (Pritchard et al. 2000), fixing *K* = 3 to match the geographic sampling regions (LOW, HIGH, NORTH), using a burn‐in of 2500 iterations followed by 10,000 MCMC repetitions. In this analysis, *K* was fixed a priori to obtain individual admixture proportions as a descriptive check of the VCA‐derived structure (our aim was to assign individuals to genetic groups and visualize the estimated admixture proportions rather than formal estimation of the ‘true’ *K*). STRUCTURE output was visualized in R with *pophelper* v.2.3.1 (Francis 2017) to plot individual admixture proportions.

To evaluate SNP associations with diapause phenotype, we used only the *phenotyped* individuals in a Bayesian framework implemented in BayPass v.2.41 (Gautier 2015), which models population structure via a scaled covariance matrix (Ω). Because BayPass operates on population‐level allele counts, we collapsed our data into two ‘populations’ defined by phenotype (obligate vs. facultative diapause) and computed, for each group, the mean VCA score as a single continuous covariate. Genotype data in VCF format were converted to BayPass‐compatible files using *reshaper_baypass.py* (Gautier 2015). We applied this identical procedure to two datasets analysed in parallel: the FULL dataset, comprising all *phenotyped* individuals from the three sampling sites (NORTH, HIGH and LOW), and the HIGH + LOW dataset, restricted to the two Central European populations in which both phenotypes occur (NORTH was excluded from this second analysis because it contributed obligate individuals only). In each case the two phenotype groups (obligate vs. facultative diapause) defined the two BayPass populations, yielding a 2 × 2 Ω matrix. BayPass' core model was first run (three independent MCMC chains per dataset, each with a different random seed) on these two allele‐count vectors to estimate the 2 × 2 Ω matrix under neutrality; the element‐wise mean Ω across the three chains was retained after confirming convergence (Spearman's *ρ* > 0.99 between chains). This fixed mean Ω was then supplied to the Importance Sampling (IS) covariate model (via the *‐omegafile* argument, ensuring identical structure correction across runs) to test for associations between SNP‐specific allele‐frequency deviations and the VCA‐score covariate. In this formulation, the response for each SNP is the pair of reference‐ and alternate‐allele counts in the obligate and facultative groups, and the explanatory variable is the corresponding mean VCA score per group. Five independent IS runs, each with a different random seed, were performed per dataset; per‐SNP Bayes Factors (BF) were summarized as the median across the five runs to reduce the influence of run‐specific stochasticity and to balance sensitivity against the false‐positive rate (Gautier et al. 2018). Association strength was reported as Bayes Factors (BF) in decibans (dB), with the median BF across runs used to reduce the influence of run‐specific variation and to balance sensitivity and false‐positive rates (Gautier et al. 2018). Five independent MCMC runs (npilot = 20, pilotlength = 200, burnin = 2,500), each with a different random seed, yielded consistent hyper‐parameter estimates. Association strength was reported as the median BF across runs, with SNPs with BF > 2 considered to have a weak association, BF > 6 a moderate association, and BF > 10 a strong association with diapause phenotype, following the conventional interpretative scale for Bayes Factors (Jeffreys 1961; Kass and Raftery 1995).

To characterize the null Bayes Factor distribution expected under population structure alone, we calibrated the BF scale empirically against pseudo‐observed datasets (PODs). Using the *simulate_baypass* function (Gautier 2015), we simulated 10,000 null SNPs under each dataset's estimated covariance (Ω) matrix and genome‐wide allele‐frequency (Beta) spectrum; because these data are generated under the population covariance alone, with no SNP‐covariate association, their BF distribution represents the values expected by chance given the structure and sample sizes of each dataset. We then ran the full IS model on the simulated data, using the same (mean) Ω and VCA‐score covariate as in the corresponding empirical analysis, and recorded the resulting null BF distribution. For each threshold we computed an empirical false‐positive rate as the proportion of null POD SNPs exceeding that threshold. For individual candidate loci we additionally computed a per‐locus empirical false‐discovery rate, defined as the expected number of null POD SNPs at or above the locus's observed BF divided by the number of observed SNPs with a BF at least as large. Both quantities were computed separately for the FULL and HIGH + LOW datasets (Figure S2).

### 2.4 Machine Learning‐Based Ranking of SNP Importance for Phenotypic Differentiation

To independently validate the predictive contribution of each SNP on determining the diapause phenotype of an individual (obligate vs. facultative diapause), we employed a supervised gradient boosting decision tree (GBDT) algorithm, implemented in LightGBM v.4.5.0 (Ke et al. 2017). Using this classifier, each SNP is assigned a quantitative importance score reflecting its contribution to predicting obligate vs. facultative diapause, analogous to effect sizes in association studies; these scores permit an ordered ranking of loci by their predictive power (Ahmed et al. 2022; Li et al. 2018; Pirmoradi et al. 2020; Silva et al. 2022), in our case the association of a SNP to the presence or absence of a diapause phenotype.

Our ML analysis proceeded in two stages: a feature‐selection stage, in which all 11,867 SNPs (referred to as *11k SNP‐set* hereafter) were ranked by importance, and a best‐model stage, in which a compact, pre‐selected feature set was used to train the final classifier. Below, we describe how genotype data are encoded, how the model is trained and tuned via cross‐validation, and how the resulting ranked list of candidate SNPs is extracted for downstream validation.

To facilitate the dimensional reduction of the SNP dataset and enable supervised learning, bi‐allelic diploid genotypes of the *phenotyped* dataset were encoded as ordinal integers. From the filtered variant call file data, genotypes were encoded as 0 for homozygous reference (0/0), 1 for heterozygous (0/1 and 1/0) and 2 for homozygous alternate (1/1), following the standard additive dosage convention that preserves the linear spacing implied by additive contributions of allelic substitutions. Notably, sites with missing data (./.) were encoded as NA. LightGBM natively treats missing values as a separate category, assigning them to the decision branch that minimizes loss at each split rather than requiring explicit imputation. It uses a histogram‐based approach and gradient‐based one‐sided sampling to efficiently process missing data, dynamically learning optimal defaults without bias. This built‐in mechanism preserves the underlying data patterns and robustness of the model, making additional encoding or imputation of missing values unnecessary (Barbier et al. 2016; Ke et al. 2017; Shi et al. 2022). Nonetheless, samples containing more than 10% missing loci were removed from the machine learning (ML) classification analysis.

In preliminary model runs, we found that modest adjustments to key hyperparameters (e.g., learning rate, number of leaves, maximum depth) produced appreciable shifts in cross‐validated precision, underscoring the classifier's sensitivity to these settings. Given this sensitivity of model accuracy to parameter tuning, we performed pre‐analytical Bayesian hyperparameter optimization using Optuna v.4.2.0 (Akiba et al. 2019). The tuning process explored a range of parameters (e.g., learning rate, number of leaves, maximum depth, regularization terms) across 2000 optimization trials, with performance estimated via 50‐fold cross validation in each trial (i.e., the dataset was randomly partitioned into 50 folds, with each fold serving once as the test set). K‐fold cross validation is crucial for feature importance ranking in GBDT models as it mitigates overfitting by evaluating the model across multiple training‐validation splits, ensuring SNP rankings are consistent and robust, and reducing the risk of spurious associations (Varma and Simon 2006). We trained LightGBM and evaluated performance under stratified k‐fold cross‐validation, preserving the obligate versus facultative‐diapause ratio (*scale_pos_weight* parameter in LightGBM) within every fold to mitigate class‐imbalance effects. The single best‐performing hyperparameter configuration identified by Optuna was fixed and used in all subsequent LightGBM analyses (Figure S3).

The mean Area Under the Receiver Operating Characteristic Curve (AUROC) score across folds was used as the objective metric to evaluate parameter performance (Roberts et al. 2024). In the feature‐selection stage, a LightGBM model was first trained on the *phenotyped* dataset using the entire *11k SNP‐set*, and the mean importance of each SNP across the 50 cross‐validation folds was recorded to produce a ranked locus list. We checked the accuracy of the trained model using different SNP‐sets to capitalize on the best SNP selection possible to determine the samples' diapause phenotypes. We tested two randomly generated genotypes with 1000 and 2500 SNPs, the top SNPs identified on the reduced‐representation genome‐wide association study (rGWAS) with different BF thresholds (BF > 2, BF > 6 and BF > 10), the top SNPs identified via ML (top 300, top 100, top 50 and top 25), and different combinations of all SNP‐sets. The best‐performing configuration combined the top 25 ML‐ranked SNPs with the rGWAS loci at BF > 6 (their union), augmented with the top three variance components (VCs) as covariates to correct for geographical stratification (Price et al. 2006). This combined feature set was then used to retrain the final (‘best’) LightGBM classifier, again under 50‐fold stratified cross‐validation, which we report as the principal model.

Additionally, to assess whether SNP rankings were driven by population structure rather than phenotypic association, we conducted a complete parallel analysis using only HIGH and LOW populations (HIGH + LOW dataset), excluding all NORTH individuals. This subset contains segregating variation for diapause within Central European populations, enabling evaluation of which SNPs differentiate phenotypes independently of possible latitudinal population structure (as NORTH was composed only of obligate diapause phenotypes). Cross‐validation, parameter optimization, model training and feature importance ranking were implemented in a custom Python pipeline using *pandas* v.2.1.4, *NumPy* v.2.1.3, *scikit‐learn* v.1.5.2 and *joblib* v.1.4.2 (Harris et al. 2020; McKinney 2010; Pedregosa et al. 2011).

### 2.5 Validation of the Predictive Model

Because cross‐validated performance can be inflated by residual population structure, we subjected the HIGH + LOW best model to three complementary validation tests, each addressing a different aspect of model validity. First, to test whether the observed performance exceeded chance, we performed a phenotype‐label permutation test (Ojala and Garriga 2010). Phenotype labels were shuffled within each population (HIGH and LOW separately) rather than globally, preserving within‐population class ratios and ensuring the test isolates phenotype signal from any residual within‐population structure. The model was retrained and re‐evaluated under the same 50‐fold cross‐validation for each of 1000 permutations, and the empirical *p*‐value was computed as the proportion of permuted AUROCs equal to or exceeding the observed value, with the (Phipson and Smyth 2010) correction to avoid a *p*‐value of zero.

Second, to test whether the phenotype signal generalizes across geography, we performed leave‐one‐population‐out (LOPO) cross‐validation (Lotterhos 2024): the model was trained on one Central European population and used to predict the other (HIGH → LOW and LOW → HIGH), with cross‐population AUROC quantifying transferability. As a positive control, the Central‐European‐trained model was then applied to the NORTH population (both the 57 *phenotyped* (obligate‐only) individuals and the NORTH *wild* individuals) where obligate diapause is expected to predominate (Schebeck et al. 2017, 2022). To distinguish within‐population segregating signal from latitudinal differentiation, we repeated the entire LOPO procedure with the FULL SNP feature set (NORTH always held out of training), allowing a direct comparison of how the two feature sets extrapolate to NORTH. We note that the FULL feature set was selected using all *phenotyped* individuals, including NORTH; consequently, its features are not fully independent of NORTH, even though NORTH individuals were excluded from model training in this comparison.

Third, to verify that confident predictions reflect genuine genomic signal rather than artefacts of model structure, we compared the distribution of confidence scores *P*(obligate) for *wild* individuals against two null datasets. A marginal‐preserving null retained each SNP's observed genotype‐frequency distribution but drew individuals independently, thereby destroying the multi‐locus genotype–phenotype association while preserving single‐locus allele frequencies; an extreme null assigned equiprobable genotypes and set covariates to zero. Mock individuals were generated to match the column identity and order of the training feature matrix, and the same trained models were applied to them. The real *wild* and null confidence‐score distributions were compared with the two‐sample Kolmogorov–Smirnov (KS) test (Kolmogorov 1933; Smirnov 1939); divergence of the real distribution from the null indicates that the model responds to genuine structure, a necessary but not sufficient condition for predictive accuracy, which is established by the permutation and LOPO tests above (Lotterhos 2024).

### 2.6 Machine Learning‐Based Phenotypic Differentiation of *Wild* Populations and a Test to Predict Outbreak Risk

Having established model reliability on these controlled datasets, we applied both the FULL and the HIGH + LOW best models to our *wild* beetle samples. For each individual we recorded the model's confidence score, *P*(obligate), and applied a 0.5 threshold to assign a binary phenotype (Ke et al. 2017; Shi et al. 2022). For each wild population we report the predicted proportion of obligate‐diapausing individuals together with a bootstrapped 95% confidence interval (2000 bootstrap resamples), and we use the resulting frequency of facultative‐diapausing beetles to infer voltinism potential and outbreak risk (see Section 3). Because one aim was to assess whether the models can separate natural populations expected to differ in diapause frequency, we further tested whether the predicted obligate proportions of the HIGH and LOW populations differed beyond sampling variation. The HIGH minus LOW difference in predicted obligate proportion was assessed with the same 2000‐resample bootstrap, taking the 2.5th to 97.5th percentiles of the between‐population difference; a 95% interval excluding zero was interpreted as a statistically distinguishable difference rather than one attributable to sampling. Because *wild* individuals were entirely absent from model fitting and tuning, these classifications constitute the independent application of the models.

### 2.7 Functional Annotation

To interpret the biological relevance of SNPs associated with diapause phenotype, we leveraged curated genome annotations and gene ontology (GO) terms from (Powell et al. 2021), transferred to our reference assembly, to functionally characterize candidate loci identified by rGWAS and ML analyses. Genome annotations were transferred to our reference genome using Liftoff v.1.6.3 (Shumate and Salzberg 2021). SNP positions were mapped against annotated gene regions, and where functional annotations were available, corresponding GO terms were recorded. This approach enabled us to link significant SNPs to putative gene functions and biological processes implicated in diapause regulation.

SNPs were mapped to genomic features based on annotation categories available in the transferred gene models, including coding sequences (CDS), exons and untranslated regions (5′ UTR and 3′ UTR). SNPs outside annotated gene boundaries were classified as intergenic. Additionally, GO terms were assigned to genes based on annotation, and for genes lacking GO, putative function was inferred from homology or literature curation. These annotations allowed us to evaluate the potential functional roles of highly associated loci in relation to known diapause mechanisms and provided a biologically meaningful foundation for linking SNP associations to putative functional consequences.

## 3 Results

### 3.1 ddRAD Sequencing and SNP Filtering

After demultiplexing and quality filtering we generated a total of 676,999,496 raw reads for 574 individuals (Table S1). We recovered 15,303 RAD loci containing 134,410 SNPs (57.79% missing sites), retaining only biallelic sites that met read depth and genotype call thresholds (Table S1). The high proportion of missing sites in the raw dataset is inherent to reduced‐representation sequencing approaches, where stochastic variation in restriction site coverage across individuals results in loci being sequenced in some but not all samples (Peterson et al. 2012). Subsequent filtering, removing loci with more than 25% missing data and pruning for linkage disequilibrium (*r* ^2^ < 0.2), reduced missing data to within acceptable limits and ensured that the final SNP‐set consisted of high‐quality, approximately independent markers. A final merged dataset of 11,867 SNPs (*11k SNP‐set*) remained after filtering out loci with more than 25% missing data and pruning for linkage disequilibrium (out of 296 *phenotyped* and 278 *wild* individuals, respectively, remained after filtering and LD pruning). The *11k SNP‐set* variant call file was split into two datasets: (1) genotypes for samples that had their diapause phenotype determined in the laboratory, that is, facultative versus obligate diapause, designated as *phenotyped* (296 individuals, comprising HIGH *n* = 120 [60 obligate, 60 facultative], LOW *n* = 119 [59 obligate, 60 facultative], NORTH *n* = 57 [57 obligate, 0 facultative]; 176 obligate and 120 facultative in total) and (2) genotypes for *wild* individuals collected from the field with no predetermined diapause phenotype (278 individuals, comprising HIGH *n* = 92, LOW *n* = 93, NORTH *n* = 93).

### 3.2 Genetic Relatedness, Population Structure and Diapause rGWAS

**FIGURE 1:**
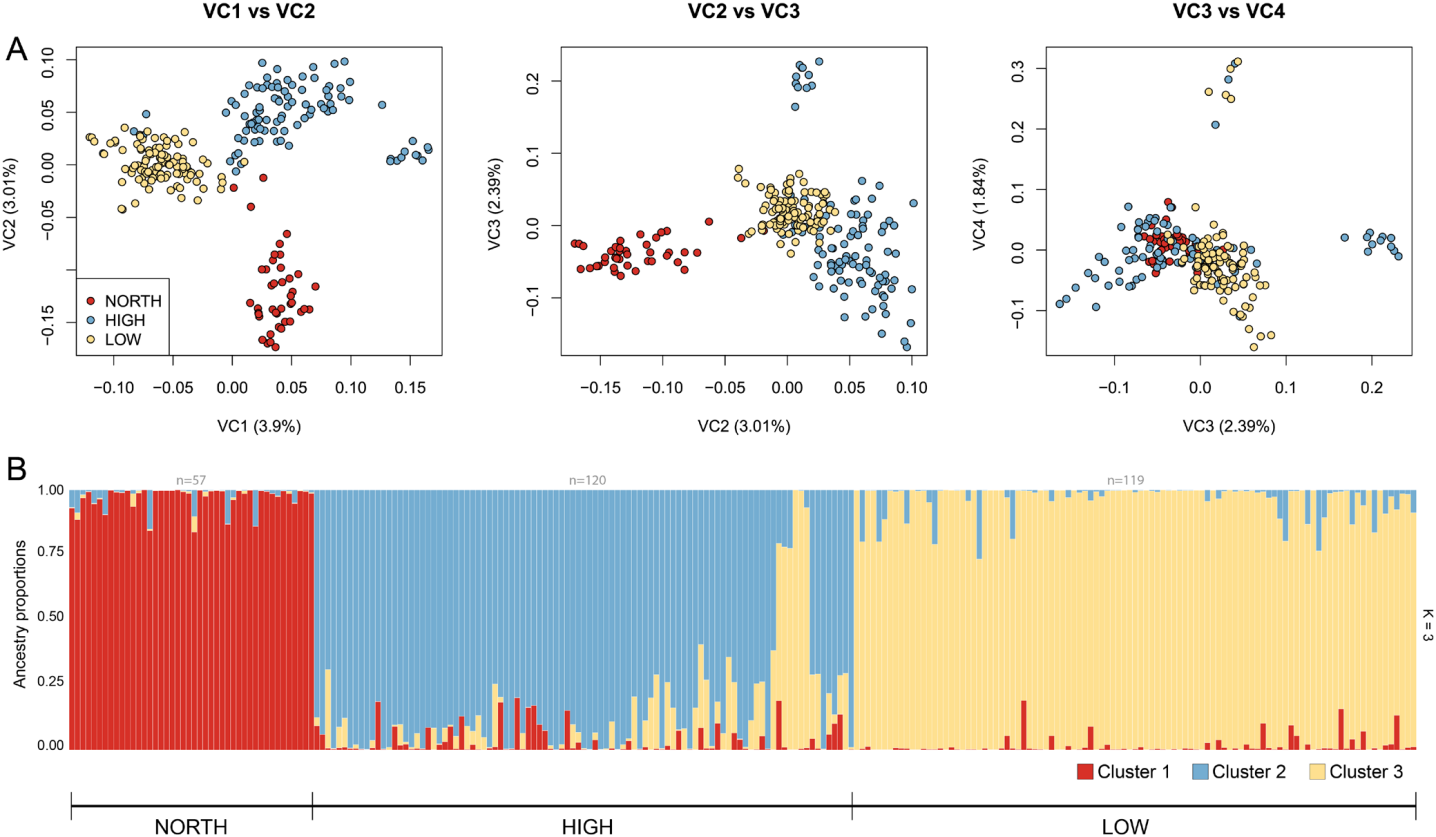
Genetic relatedness and population structure among *phenotyped Ips typographus* individuals. (A) Variation component (VC) analysis showing sample distribution along the first four components. The first three VCs reflect strong geographic structuring, while subsequent components account for progressively less variation. Within‐population sub‐clusters reflect shared ancestry and gene flow between geographically proximate sites, particularly between HIGH and LOW individuals, and do not correspond to diapause phenotype. (B) Ancestry proportions inferred with STRUCTURE, assuming three genetic clusters. Bars represent individual beetles coloured by their proportional ancestry and ordered by site. Populations correspond to three geographic sites: NORTH (SW‐Vindeln), HIGH (AT‐Gesäuse) and LOW (AT‐Prinzersdorf).

Variation component analysis of the genomic relationship matrix identified 265 variation components (VCs) capturing the total genetic variance. The first three VCs explained the strongest geographical signal (9.3%), while higher‐order components accounted for progressively smaller fractions. VCA scores revealed clear geographic clustering, separating individuals from NORTH, HIGH and LOW (Figure 1A). A small subset of individuals showed intermediate VCA values, suggesting partial admixture or overlapping genetic backgrounds, particularly among the LOW and HIGH populations. When *K* was set equal to three, STRUCTURE also identified clusters that corresponded with the NORTH, HIGH and LOW collection sites, recovering three genetic clusters, and also suggested admixture in some HIGH population individuals (Figure 1B).

Under the BayPass importance sampling (IS) covariate model, mean VCA scores served as a single quantitative covariate contrasting obligate vs. facultative diapause phenotypes. Bayes Factors (BF) were highly consistent across five MCMC replicates (Table S2). In the FULL dataset, of the *11k SNP‐set* evaluated, 76 SNPs exhibited a weak (BF > 2) association with diapause phenotype, 23 showing moderate support (BF > 6), and nine exceeded BF > 10 and were considered as having a strong association (Table 1). The strongest association by a wide margin was a SNP occurring on *I. typographus* genome contig *IpsContig35* (locus 15284, position 32) annotated to juvenile hormone esterase (JHE) function, with a median BF of 59.9, more than twice that of the next‐ranked locus (see Table S2 for full annotation reference). Of the remaining eight SNPs strongly associated with diapause phenotype (BF > 10), several mapped to genes with putative functions in transcriptional regulation, signal transduction and stress response. The complete list of SNPs and their putative functional annotations is presented in Table 1.

**TABLE 1:** SNPs associated with diapause phenotype in the FULL rGWAS analysis (BayPass median Bayes Factor > 6), ranked by Bayes Factor.

CHROM	SNP	Gene name	Description	GO IDs	BF median
IpsContig35	locus 15284 pos32	Ityp17178	Juvenile hormone esterase (JHE)	F:GO:0016787	59.915
LG04	locus 10700 pos70	Ityp09718	Hypothetical protein YQE_03602, partial	F:GO:0003824; F:GO:0005516; C:GO:0005886; P:GO:0005977	23.666
LG02	locus 6422 pos76	—	Not annotated	—	18.701
LG11	locus 3131 pos10	Ityp21900	Hypothetical protein D910_02874	F:GO:0005044; P:GO:0006897; C:GO:0016021	14.732
LG04	locus 10181 pos72	Ityp17571	PREDICTED: uncharacterized protein LOC108573940	—	14.061
LG05	locus 11709 pos30	Ityp08787; Ityp08786	PREDICTED: murinoglobulin‐2‐like/tetratricopeptide repeat protein 37	—	12.421
LG12	locus 4042 pos40	Ityp04943	Epimerase, partial	P:GO:0006629; C:GO:0016021; P:GO:0055114; F:GO:0080019; F:GO:0102965	10.805
LG03	locus 8848 pos27	—	Not annotated	—	10.466
LG15	locus 4827 pos57	Ityp18712	Eukaryotic translation init. factor 4 gamma 3‐like isoform X1	—	10.195
LG06	locus 12372 pos51	Ityp04143	Hypothetical protein D910_06717	P:GO:0006811; C:GO:0016020; P:GO:0055085	9.315
LG05	locus 11638 pos9	—	Not annotated	—	8.908
LG01	locus 989 pos71	Ityp09097	Protein tamozhennic	—	8.480
LG16	locus 5255 pos120	Ityp13190	Hypothetical protein YQE_02493, partial	—	8.464
LG01	locus 2489 pos78	Ityp05778	PREDICTED: uncharacterized protein LOC109538932	—	8.174
LG12	locus 3855 pos36	Ityp14600	Hypothetical protein YQE_10721, partial	—	7.647
LG09	locus 15047 pos40	Ityp07880	—	—	7.454
LG02	locus 7586 pos6	Ityp01245	Nucleolar GTP‐binding protein 2	F:GO:0005525; C:GO:0005730	7.330
LG02	locus 6367 pos72	Ityp00005	U6 snRNA‐associated Sm‐like protein LSm1	F:GO:0000339; C:GO:0000932; P:GO:0000956; P:GO:0006397	7.230
LG03	locus 9158 pos50	—	Not annotated	—	7.015
LG02	locus 6689 pos18	Ityp00286	Uncharacterized protein LOC111419767	—	6.999
LG12	locus 4031 pos61	Ityp07311	Cytochrome P450 6k1‐like isoform X1	F:GO:0004497; F:GO:0005506; C:GO:0016021; F:GO:0016705; F:GO:0020037; P:GO:0055114	6.939
LG09	locus 14925 pos65	Ityp08057	Protein tramtrack, beta isoform‐like isoform X1	—	6.412
LG06	locus 12377 pos56	—	Not annotated	—	6.063

*Note:* Gene names, descriptions and gene ontology (GO) terms were extracted from Powell et al. (2021). Linkage groups or contig names (CHROM) follow Nadachowska‐Brzyska et al. (2026). The complete list of all *11k SNP‐set* evaluated is provided in Table S2.

**FIGURE 2:**
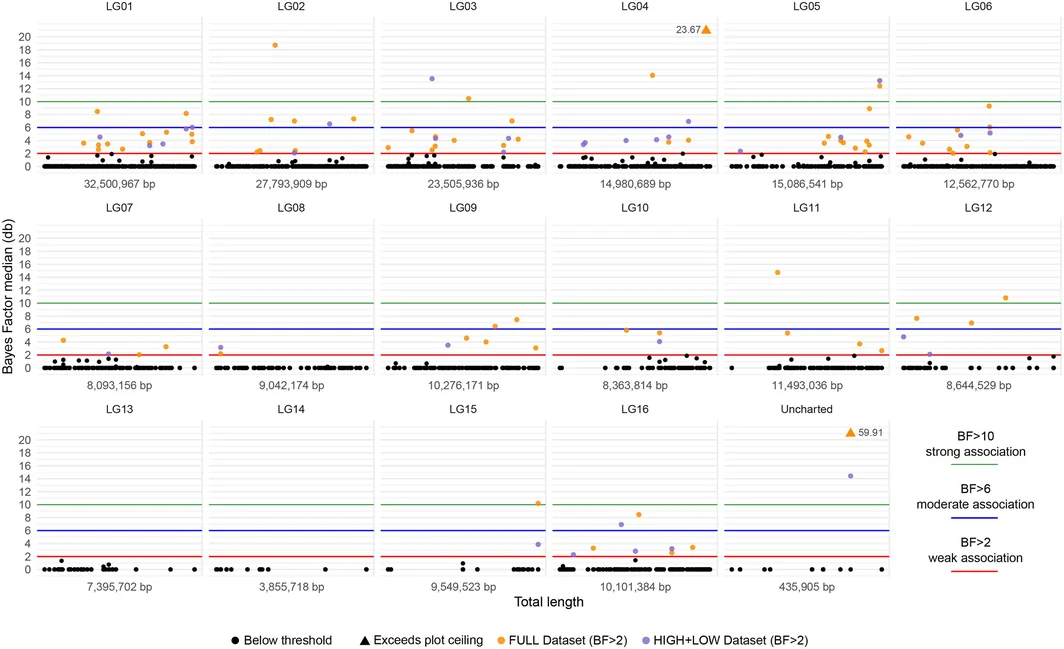
Results of BayPass analysis showing the strength of SNP associations with the diapause phenotype across the *Ips typographus* genome. SNPs (dots) are ranked by their median Bayes Factor (BF) across five independent BayPass runs. The *x*‐axis indicates genomic position organized by linkage groups (LGs) likely corresponding to the bark beetle chromosomes (Nadachowska‐Brzyska et al. 2026), and the *y*‐axis displays Strength of Association (Bayes Factor). Horizontal lines represent thresholds for association: BF > 2 (red) indicates weak association, BF > 6 (blue) moderate association and BF > 10 (green) strong association. FULL dataset refer to the model including NORTH, HIGH and LOW populations; HIGH + LOW dataset includes only Central Europe populations.

We repeated the rGWAS on the HIGH + LOW dataset, in which both phenotypes occur within each Central European population and NORTH (obligate‐only) is excluded. As expected for a within‐population contrast, fewer and weaker associations were recovered: 34 SNPs at BF > 2, seven at BF > 6 and three at BF > 10. Notably, the same JHE SNP *IpsContig35* (locus 15284, position 32) remained the top‐ranked hit (median BF = 14.4), and a mitochondrial solute‐carrier SLC25 family; *LG03* (locus 8423, position 38) ranked second (BF = 13.5). Only two SNPs were shared between the FULL and HIGH + LOW moderate‐association sets (BF > 6): the JHE locus and *LG05* (locus 11709, position 30), indicating that most associations are specific to one analysis front (Figure 2; Table 1).

We calibrated these thresholds against pseudo‐observed datasets (PODs) simulated under each dataset's estimated covariance matrix and allele‐frequency spectrum (Figure S2). In the FULL dataset, 0.43% and 0.23% of null SNPs reached BF > 6 and BF > 10, respectively; the corresponding rates for the HIGH + LOW dataset were 0.34% and 0.08%. Because these rates apply across the *11k SNP‐set* panel, we treat the BF > 6/10 categories as a candidate‐selection criterion rather than as a per‐SNP control of the genome‐wide false‐discovery rate, and we assessed each nominated locus directly against the POD null. Only the JHE locus lay entirely beyond that null: no simulated SNP reached its Bayes factor (59.9 vs. a POD maximum of 24.6 dB), corresponding to an empirical false‐discovery rate of nearly 0 and identifying JHE as the single rGWAS association decisively robust to population structure. Support fell away sharply thereafter, the next‐ranked locus already carried an empirical false‐discovery rate of 0.59 (BF = 23.7), so the remaining threshold‐passing loci are retained as candidates for downstream analysis rather than as individually significant associations. In the HIGH + LOW dataset no locus exceeded the POD null (top BF = 14.4 vs. a POD maximum of 17.9 dB), consistent with the reduced power of the within‐population contrast; these candidates are interpreted below in light of their convergence with the machine‐learning model (Figure S2).

### 3.3 Validation of the Predictive Model

To assess whether the HIGH + LOW model's cross‐validated performance exceeded chance, we performed a phenotype‐label permutation test with 1000 shuffles (Phipson and Smyth 2010). None of the 1000 permuted models reached the observed AUROC of 0.8783 (null mean = 0.497, SD = 0.059; empirical *p* = 0.001), confirming that the signal recovered by the classifier is not attributable to within‐population class imbalance or stochastic variation (Figure 6A).

Leave‐one‐population‐out (LOPO) cross‐validation further demonstrated that phenotype associations generalize across geography within Central Europe. Training on HIGH and predicting LOW, and vice versa, yielded mean cross‐population AUROCs of 0.7835 and 0.7494, respectively (mean = 0.7665; Figure 6B), well above the 0.5 null. When the Central‐European‐trained model was applied to the NORTH population, where obligate diapause is expected to predominate, 68.4% of *phenotyped* NORTH individuals and 49.5% of *wild* NORTH individuals were classified as obligate diapausing using HIGH + LOW features alone (Figure 6C). By contrast, repeating the LOPO procedure with the FULL feature set (which includes JHE; NORTH always withheld from training) yielded near‐complete classification of the NORTH *phenotyped* individuals as obligate (98.25%) and classified 84.95% of NORTH *wild* individuals accordingly. We note that the FULL feature set was selected using all 296 *phenotyped* individuals, including NORTH; consequently, the FULL‐feature NORTH result is not fully independent of NORTH and should be interpreted as corroborating, rather than independent, evidence.

Null calibration confirmed that the model responds to genuine genomic signal. Confidence scores *P*(obligate) for wild individuals diverged significantly from the extreme null (KS test *p* = 3.1 × 10^−5^ for the HIGH + LOW model; *p* = 9.5 × 10^−6^ for the FULL model). However, HIGH + LOW wild scores did not differ significantly from the marginal‐preserving null (KS *p* = 0.34), consistent with a model that captures polygenic structure rather than dominant single‐locus signal; individual‐level confidence scores for HIGH + LOW *wild* predictions should therefore be interpreted as population‐level estimates rather than deterministic individual classifications (Figure 5C).

### 3.4 ML Benchmarking and Feature Importance Scores

We used a supervised gradient‐boosted decision‐tree model (LightGBM) to predict diapause phenotype from SNP genotypes, evaluating performance with AUROC, which quantifies how well the model discriminates between facultative and obligate individuals (0.5 = random, 1.0 = perfect separation).

**FIGURE 3:**
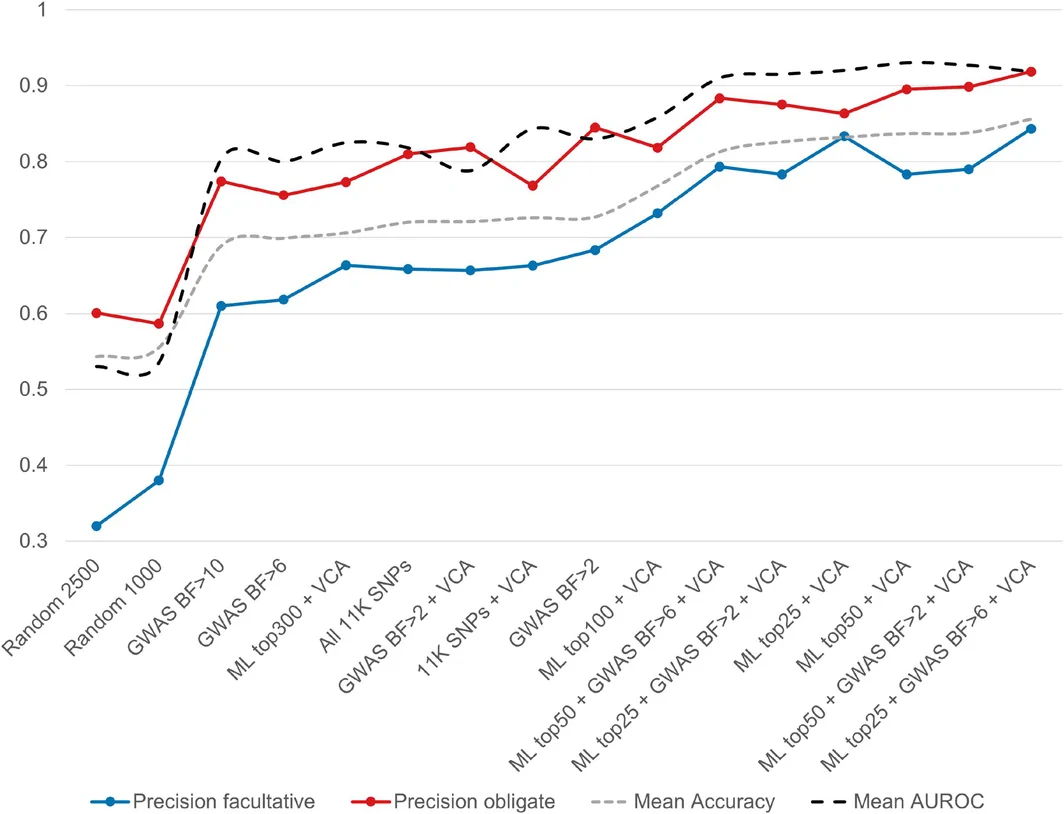
Model benchmark and SNP set selection for LightGBM training. Classification performance is compared across different SNP sets derived from machine learning (ML) feature ranking, variance component analysis (VCA) and reduced‐representation genome‐wide association study (rGWAS) filtering based on Bayes Factor. Metrics include mean accuracy, Area Under the Receiver Operating Characteristic curve (AUROC) and class‐specific precision for obligate and facultative diapause phenotypes.

Among all configurations tested, the model combining the top 25 ML‐ranked SNPs with loci showing BF > 6 in the rGWAS, together with the top three VCs to control for structure, achieved the best predictive performance (AUROC = 0.9225; mean accuracy = 0.896; Figure 3), yielding a combined feature set of 41 SNPs (25 ML‐ranked loci and 23 rGWAS loci at BF > 6, of which 7 were shared, plus 3 VCs). This configuration (ML top25 + GWAS BF > 6 + VCA) provided the greatest reliability in distinguishing obligate (accuracy = 0.9183) and facultative (accuracy = 0.8433) diapause phenotypes and was therefore retained as the principal classifier. The LightGBM classifier trained with the top 50 SNPs from the *11k SNP‐set* combined with the top three VCs reached a similar maximum cross‐validation AUROC of 0.93 (mean accuracy = 0.837). Bayesian hyperparameter tuning improved the baseline AUROC from 0.67 to 0.86 (Figure S3), indicating a substantial gain in the model's ability to separate phenotypes across all probability thresholds (Figure S3).

The top 25 SNPs identified through ML classification represent loci that most effectively differentiate between facultative and obligate diapause phenotypes in *I. typographus* individuals. Across the two best‐model feature sets (FULL and HIGH + LOW), 13 SNPs were shared, indicating a common core of diapause‐associated loci recovered by both analyses (Table 2). Notably, only five SNPs overlapped between the BayPass rGWAS (BF > 10) and the ML top 25: represented by *I. typographus* genome contig *IpsContig35* (locus 15284, position 32), *LG04* (locus 10700, position 70), *LG04* (locus 10181, position 72), *LG05* (locus 11709, position 30) and *LG15* (locus 4827, position 57). The consistent identification of *IpsContig35* (locus 15284, position 32) as the top SNP across all ML runs, with an importance score exceeding twice that of the next ranked feature (Table 2), highlights that variation at this locus (i.e., JHE function) is strongly associated with variation in diapause phenotypes.

**TABLE 2:** Top 25 SNPs associated with diapause phenotype in the FULL analysis, ranked by LightGBM feature importance.

CHROM	SNP	Gene name	Description	GO IDs	ML importance
IpsContig35	locus 15284 pos32	Ityp17178	Juvenile hormone esterase (JHE)	F:GO:0016787	2362.98*
LG04	**locus 10181 pos72**	**Ityp17571**	**PREDICTED: uncharacterized protein LOC108573940**	—	919.79*
LG12	locus 4031 pos61	Ityp07311	Cytochrome P450 6k1‐like isoform X1	F:GO:0004497; F:GO:0005506; C:GO:0016021; F:GO:0016705; F:GO:0020037; P:GO:0055114	697.24*
LG03	**locus 8423 pos38**	**Ityp13040**	**Solute carrier family 25 member 46‐like isoform X1**	**C:GO:0005741; C:GO:0016021; P:GO:0090149**	**629.87**
LG09	**locus 14872 pos73**	**Ityp12136**	**PREDICTED: tankyrase**	—	**464.42**
LG04	locus 10700 pos70	—	Not annotated	—	442.40*
LG13	**locus 4199 pos32**	**Ityp15231;** **Ityp15230**	**Not annotated**	—	**435.80**
LG03	**locus 8448 pos167**	—	**Not annotated**	—	**416.04**
LG10	**locus 2829 pos156**	**Ityp12669**	**Not annotated**	—	**404.30**
LG10	locus 2833 pos73	—	Not annotated	—	387.58
LG03	**locus 7782 pos25**	—	**Not annotated**	—	**385.88**
LG06	locus 12132 pos24	Ityp10778	PREDICTED: uncharacterized protein locus 109538902	—	370.95
LG06	**locus 12379 pos63**	—	**Not annotated**	—	**357.17**
LG05	**locus 11709 pos30**	**Ityp08787;** **Ityp08786**	**Not annotated**	—	347.44*
LG02	locus 7586 pos8	Ityp01245	Nucleolar GTP‐binding protein 2	F:GO:0005525; C:GO:0005730	308.64
LG01	**locus 2489 pos78**	**Ityp05778**	**Not annotated**	—	293.14*
LG02	locus 5853 pos70	Ityp13724	Not annotated	—	278.36
LG05	locus 10861 pos94	—	Not annotated	—	242.17
LG07	**locus 13390 pos12**	**Ityp16724**	**Transposable element Tc3 transposase**	**F:GO:0003964; P:GO:0006278**	**240.04**
LG01	locus 752 pos67	—	Not annotated	—	228.57
LG02	locus 7749 pos58	—	Not annotated	—	224.33
LG03	locus 8313 pos27	Ityp10919; Ityp01989	PREDICTED: uncharacterized protein locus 109541849/UPF0472 protein C16orf72 homologue isoform X2	C:GO:0016021	207.69
LG11	locus 3469 pos98	Ityp05181	Arylsulfatase B‐like	—	181.68
LG02	**locus 7659 pos116**	**Ityp01309**	**Not annotated**	—	**181.37**
LG15	**locus 4827 pos57**	**Ityp18712**	**Eukaryotic translation initiation factor 4 gamma 3‐like isoform X1**	—	179.24*

*Note:* Gene names and gene ontology (GO) terms were obtained from (Powell et al. 2021). Linkage groups or uncharted contig names (CHROM) as defined in (Nadachowska‐Brzyska et al. 2026). Asterisk mark SNPs also recovered by rGWAS (BF > 6). Loci in bold mark the 13 SNPs shared between the FULL and HIGH + LOW ML top25 rankings. The complete list of all *11k SNP‐set* evaluated is provided in Table S2.

Beyond these shared loci, the remaining SNPs among the ML top 25 are identified as differentiating facultative and obligate diapause phenotypes. These SNPs are associated with genes that cluster into functional categories relevant for diapause regulation, including protein synthesis and degradation, membrane transport, signal transduction and hormonal metabolism. We interpret the small locus‐level overlap as the expected outcome of different optimization targets in BayPass versus LightGBM (see Section 4).

The HIGH + LOW analysis achieved an AUROC of 0.8783 (95% CI [0.50, 1.00]; mean accuracy = 0.766), using a combined feature set of 29 SNPs (25 ML‐ranked loci and 7 rGWAS loci at BF > 6, of which 3 were shared, plus 3 VCs), confirming that the model can distinguish diapause phenotypes using only individuals from populations with segregating variation. Comparison of SNP rankings between the FULL and HIGH + LOW datasets revealed two distinct patterns (Table S2): SNPs such as *LG03* (SLC25 family, locus 8423; rank 4 vs. 1) and LG15 (eIF4G, locus 4827; rank 25 vs. 12) showed consistent high rankings across both datasets, representing within‐population signals. In contrast, *IpsContig35* (JHE, locus 15284, position 32; rank 1 vs. 82) ranked substantially lower in HIGH + LOW, indicating that its top ranking in the complete dataset is associated with differentiation of NORTH from Central European populations. The reference allele (G) at this locus was completely fixed in the NORTH population (frequency = 1.0) compared to HIGH (0.14) and LOW (0.08), consistent with strong directional selection for obligate diapause at high latitudes.

### 3.5 Functional Annotation

For the SNPs from the rGWAS analysis with a weak association with diapause phenotype (*n* = 76; BF > 2), 44 were functionally annotated, of which 18 are located within coding regions, and four hit putative regulatory untranslated regions (UTRs), 32 intergenic. Among the top significant SNPs with moderate association (*n* = 23; BF > 6), 13 hit gene models and seven hit coding regions. Likewise, among the 25 most informative SNPs identified by the ML model (non‐zero importance score), 14 were functionally annotated, with 7 hits in coding regions and one hit on UTRs (Table S2).

### 3.6 Machine Learning‐Based Phenotypic Prediction of Wild Populations

**FIGURE 4:**
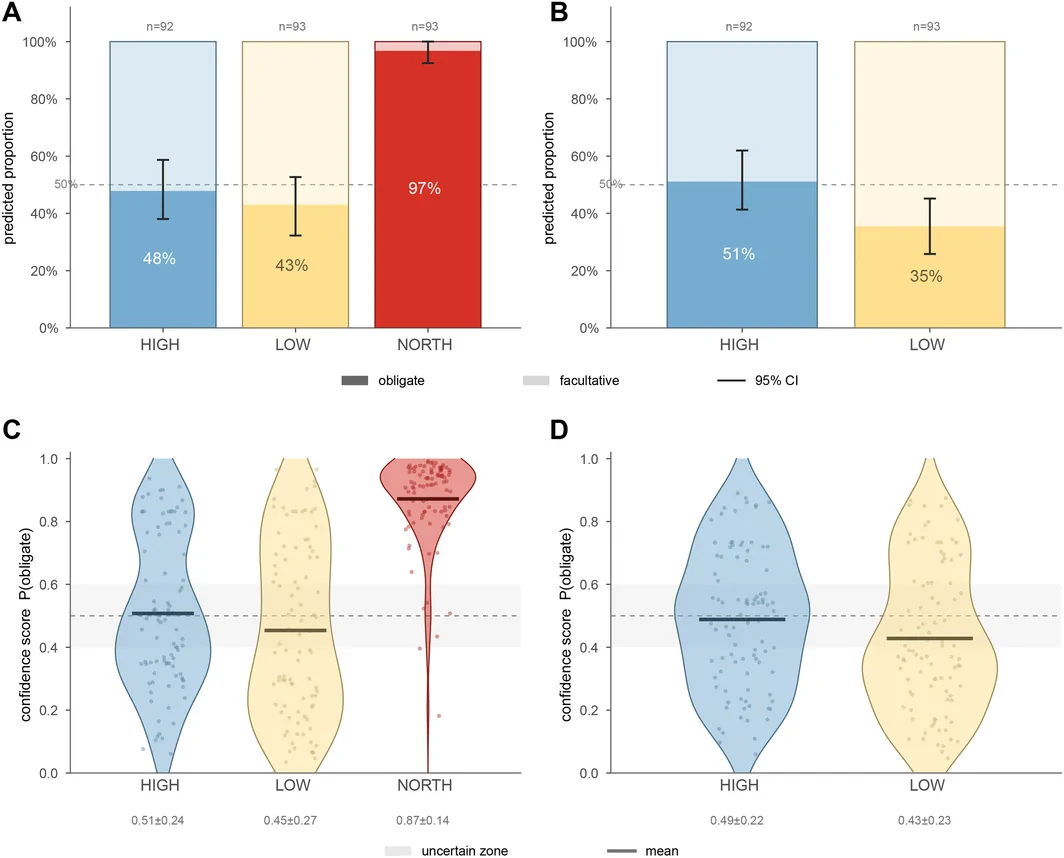
Diapause phenotype predictions for *wild Ips typographus* across three populations. Trained LightGBM models were applied to *wild* individuals from the high‐altitude (HIGH), low‐altitude (LOW) and northern (NORTH) populations on the FULL and the HIGH + LOW models. Within each bar, the predicted obligate fraction is shown as the solid fill and the facultative fraction as the pale fill. (A) FULL‐model predicted composition; error bars are bootstrapped 95% confidence intervals on the obligate proportion, and the dashed line marks 50%. (B) HIGH + LOW‐model predicted composition. (C, D) Distributions of per‐individual confidence scores, *P*(obligate), for the FULL (C) and HIGH + LOW (D) models; points are individuals, the horizontal bar is the population mean, the dashed line is the 0.5 classification threshold, and the shaded band marks the uncertain zone (0.4–0.6).

**FIGURE 5:**
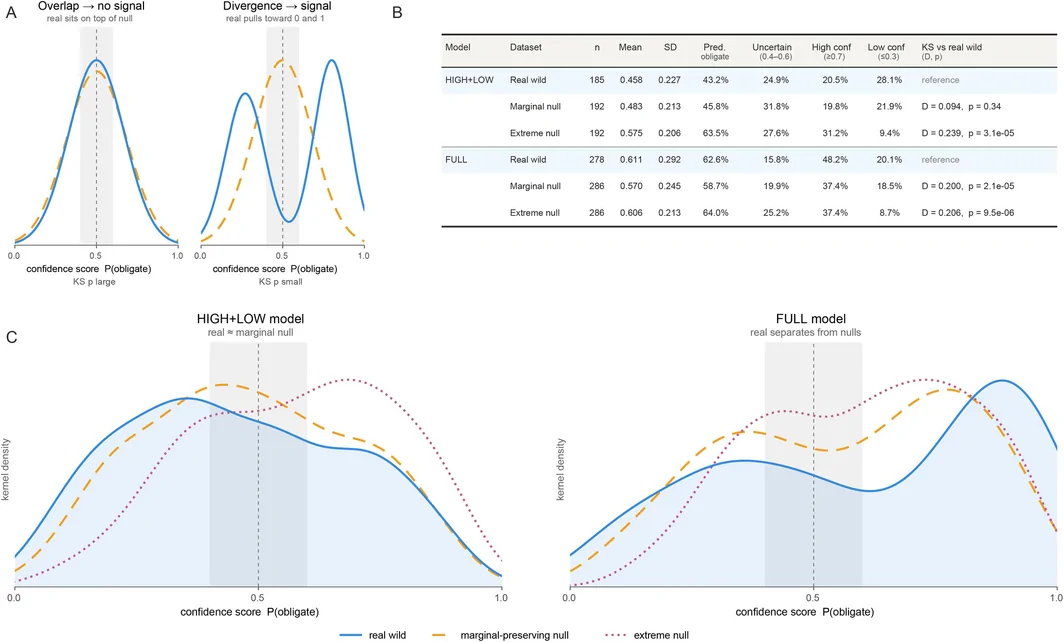
Null calibration of *wild* diapause predictions. Confidence‐score distributions for *wild* individuals were compared against two null models to test whether predictions reflect genuine genomic signal. The marginal‐preserving null retains each SNP's allele frequency but destroys the multi‐locus genotype–phenotype association; the extreme null assigns equiprobable genotypes. (A) Schematic of the test logic: When the real distribution overlaps the null (large Kolmogorov–Smirnov [KS] *p*) the model carries no signal beyond the null; when the real distribution diverges toward 0 and 1 (small KS *p*) the model resolves genuine structure. (B) Summary statistics for the HIGH + LOW and FULL models across *wild*, marginal‐preserving and extreme datasets, with the KS statistic (*D*‐value and *p*‐value of each null relative to the *wild* distribution). (C) Kernel density of confidence scores, *P*(obligate), for *wild* individuals (blue, shaded), the marginal‐preserving null (dashed) and the extreme null (dotted); shaded band marks the uncertain zone (0.4–0.6).

Both the FULL and the HIGH + LOW best models were applied to the 278 *wild*, non‐phenotyped *I. typographus*. The FULL model predicted 47.8% of HIGH *wild* individuals, 43.0% of LOW *wild* individuals, and 96.8% of NORTH *wild* individuals as obligate diapausing (bootstrapped 95% CIs reported in Figure 4A,C). The HIGH + LOW model predicted 51.1% of HIGH and 35.5% of LOW *wild* individuals as obligate diapausing (Figure 4B,D). A bootstrap comparison of the two Central European populations (2000 resamples) showed that the HIGH + LOW model resolves a significant difference in predicted obligate frequency between them (HIGH − LOW = +15.6 percentage points; 95% CI [+1.6, +29.7], excluding zero; two‐sided *p* = 0.029), in the direction expected from elevation, whereas the FULL model did not separate the populations (+4.8 points; 95% CI [−9.3, +18.8]; *p* = 0.52). Removing the latitudinal signal thus makes the within‐population model more sensitive to the elevational difference in diapause frequency. Because the HIGH + LOW model does not include NORTH‐associated signal (JHE), its confidence‐score distributions for wild individuals should be interpreted as population‐level frequency estimates rather than deterministic individual classifications, consistent with the null‐calibration results (Figure 5).

## 4 Discussion

Diapause is a fundamental adaptive strategy that enables insects to synchronize their life cycles with seasonal environments. Across taxa, this trait is characterized by complex genetic architectures that regulate the successive phases of the diapause program (Denlinger 2022; Koštál et al. 2017; Ragland et al. 2010). Diapausing insects arrest or decelerate development and reproduction, suppress metabolism, accumulate energy stores and elevate stress responses, changes that are reflected in gene expression and metabolic activity (Dowle et al. 2020; Koštál et al. 2017). Our study resolved the genomic basis of diapause polymorphism, that is, obligate versus facultative phenotypes, in *I. typographus*. Accurate phenotypic prediction indicates that this polymorphism is polygenic, regulated by many loci of small to moderate effect rather than by one or a few large‐effect variants, consistent with broader evidence across insects (Ragland et al. 2019). The implicated variants map to genes spanning hormonal regulation, lipid metabolism, stress responses, oogenesis, transcription, translation, signal transduction, membrane transport, protein degradation and photoperiodic perception (Ragland et al. 2019), indicating that diapause in *I. typographus* is governed by coordinated interactions among endocrine, metabolic, reproductive, photoperiod‐sensing and cellular‐homeostasis pathways. Because *phenotyped* individuals were reared under controlled, standardized temperature and photoperiod (Schebeck et al. 2022), the scored phenotypes reflect intrinsic genetic predisposition rather than environmentally induced variation, minimizing a key confounder in genotype–phenotype association of plastic traits.

A central feature of our results is that neither the rGWAS nor the ML analysis alone recovered a comprehensive set of diapause‐discriminating loci; the most robust signal emerged from their intersection. BayPass and LightGBM optimize different objective functions on data aggregated at different levels, so modest locus‐level overlap is expected rather than concerning. BayPass models phenotype‐defined allele‐count vectors with covariance correction and a VCA covariate, ranking SNPs by their marginal association after accounting for structure (Gautier 2015), whereas LightGBM is trained on individual genotypes and selects a sparse feature subset that maximizes out‐of‐sample discrimination by exploiting conditional, non‐linear effects while down‐weighting redundant markers (Ke et al. 2017). Accordingly, seven of the 25 most important ML features also exceeded BF > 6 in the FULL rGWAS, five of them BF > 10 (Table 2), and relaxing the threshold increased convergence further, indicating that both procedures detect the same underlying signal but rank its representatives differently. Loci recovered by both methods therefore carry the strongest evidential support, while ML‐unique features likely capture conditional effects with weak marginal association, and rGWAS‐unique loci remain biologically informative even when redundant for classification. This complementarity in the analysis is also practical: the classifier combining ML‐ranked features with rGWAS‐supported loci and VCA covariates achieved the best cross‐validated performance (AUROC = 0.9225; Figure 3), outperforming either source alone.

The clearest case of convergent support is juvenile hormone esterase (JHE; *IpsContig35*, locus 15284), the top‐ranked locus in both the FULL rGWAS (median BF = 59.9, more than twice the next locus; Table 1) and the FULL ML model (highest feature importance; Table 2). JHE is a well‐established regulator of diapause across insects, modulating juvenile‐hormone titres and thereby reproductive status and diapause expression (Denlinger 2002, 2022; Izadi 2025; Koštál et al. 2017; Ma et al. 2021; Zhou et al. 2022), so its emergence as the strongest signal provides convergent, independent evidence for its candidacy rather than a spurious association. Its behaviour across analyses, however, requires careful interpretation. When NORTH is excluded and the contrast is restricted to the within‐population, facultative‐versus‐obligate comparison of Central Europe (HIGH + LOW), JHE still passes the candidate threshold (median BF = 14.4) but is not a robust within‐population signal, consistent with the POD calibration, no HIGH + LOW locus clears the null, and it contributes almost nothing to ML discrimination, where a mitochondrial solute‐carrier locus leads instead (Table S2). This dichotomy is explained by allele frequencies: the reference allele at the JHE SNP is effectively fixed in NORTH (frequency = 1.0) but segregates at low frequency in HIGH (0.14) and LOW (0.08). Because of a locus's contribution to within‐population variance scales with 2pq, JHE can be a genuine diapause‐regulating locus yet contribute little to local discrimination (i.e., nothing in NORTH, where it is fixed, and little in Central Europe, where its variant is rare). We therefore read JHE not as an alternative to a diapause role but as that role expressed across spatial scales: near‐fixed in the north, consistent with directional selection for obligate diapause, and segregating too rarely in Central Europe to drive the local facultative‐obligate switch, which instead rests on other loci (e.g., SLC25). This scale‐dependent partitioning reconciles its dominant rank in the FULL dataset with its limited within‐population (HIGH + LOW) predictive contribution.

Complementing this population‐level signal, several loci behave as robust within‐population predictors. A mitochondrial solute‐carrier protein (SLC25 family; *LG03*, locus 8423) is the leading ML feature in the HIGH + LOW analysis and is independently supported by the HIGH + LOW rGWAS (BF = 13.5, the second‐strongest hit), even though it shows little marginal association in the FULL rGWAS, precisely the profile expected of a within‐population signal that the NORTH‐dominated complete analysis does not emphasize. Its identity is consistent with the metabolic adjustments and energy conservation that characterize diapause (Hahn and Denlinger 2011). Among the loci shared between the FULL rGWAS and ML analyses, cytochrome P450 6k1‐like (*LG12*, locus 4031; BF = 6.9) aligns with the role of P450 enzymes in juvenile‐hormone and ecdysteroid metabolism during diapause (Tian et al. 2021), and eukaryotic translation initiation factor 4 gamma (eIF4G; *LG15*, locus 4827; BF = 10.2) is consistent with translational control during diapause maintenance (Ragland et al. 2011), though its low ML ranking indicates a contributory rather than a primary predictive role. Together these loci underscore the polygenic and functionally diverse architecture of diapause in *I. typographus*.

**FIGURE 6:**
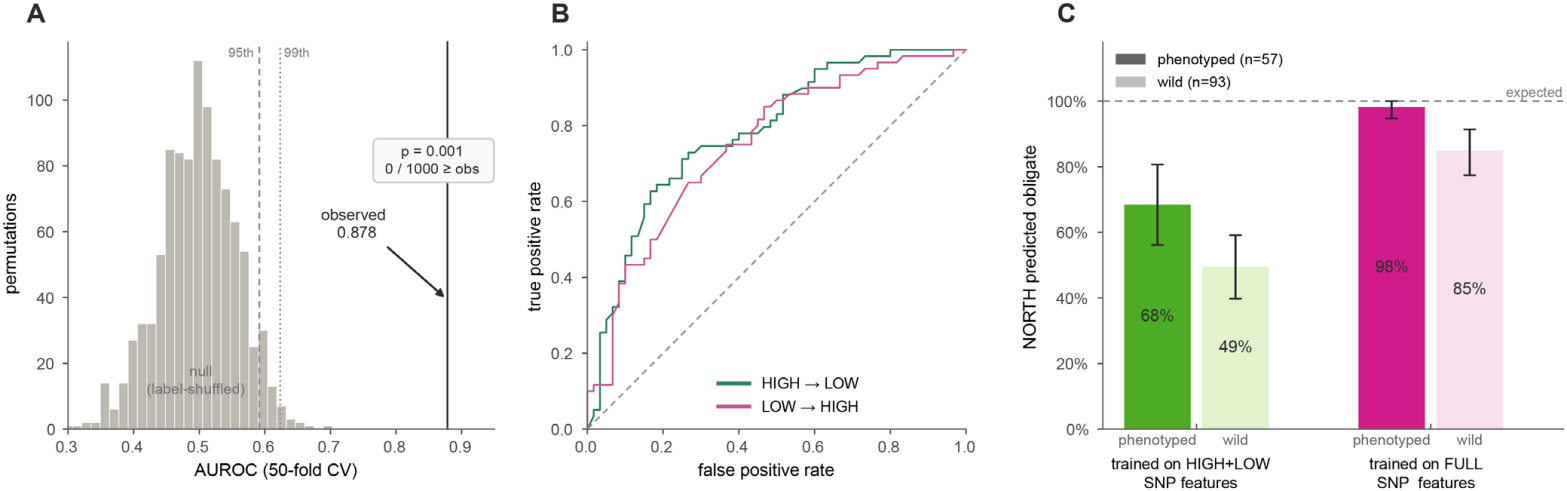
Model validation. (A) Permutation test for the HIGH + LOW model: Distribution of AUROC values from 1000 within‐population phenotype‐label permutations (50‐fold cross‐validation; null mean 0.50); dashed and dotted lines mark the 95th and 99th percentiles of the null. (B) Leave‐one‐population‐out (LOPO) cross‐population ROC within Central Europe using HIGH + LOW SNP features: training on HIGH and testing on LOW (AUC = 0.78) and the reverse (AUC = 0.75); the diagonal denotes random. (C) Generalization of LOPO to NORTH population as a positive control (expected ~100% obligate). Bars show the predicted obligate proportion (bootstrapped 95% CI) for models trained on the HIGH + LOW SNP features versus the model trained on FULL SNP features, evaluated on NORTH *phenotyped* and NORTH *wild* individuals.

The discrimination achieved by our models is not a statistical artefact. A permutation test, in which phenotype labels were shuffled, yielded an observed AUROC of 0.878 against a null distribution centred near chance (mean = 0.497), with no permutation reaching the observed value (*p* = 0.001; Phipson and Smyth 2010; Figure 6). Null calibration further showed that confidence scores for wild individuals diverge from label‐shuffled and structure‐preserving null distributions, confirming that the model responds to genuine genomic structure rather than noise (Figure 5); this is a necessary but not sufficient condition for predictive validity, which the permutation and cross‐population analyses establish (Lotterhos 2024). The within‐population model trained without NORTH (HIGH + LOW) also discriminated phenotypes well (AUROC = 0.8783), although its confidence interval is wide (0.50, 1.00) owing to the small number of individuals per cross‐validation fold. We emphasize that the lower performance of the HIGH + LOW model relative to the complete dataset (AUROC = 0.9225) reflects this reduced sample size together with the absence of the large between‐population (latitudinal) allele‐frequency differences that the FULL model additionally exploits, not a weaker or more ‘subtle’ phenotype. The facultative‐obligate contrast is a genuine binary phenotype assayed under controlled conditions (Schebeck et al. 2022), and its within‐population genetic signal is real (permutation *p* = 0.001), if necessarily harder to resolve than the population‐level differentiation that dominates the complete dataset.

Leave‐one‐population‐out (LOPO) cross‐validation clarified this distinction. Within Central Europe, the two feature sets performed similarly (mean AUROC ~0.77 for HIGH + LOW features and ~0.75 for FULL features; Figure 6; Figure S4), indicating that the additional features in the FULL model do not simply improve local phenotype discrimination. The difference emerged when models were extrapolated to the NORTH population. HIGH + LOW features, which capture within‐population segregating variation, predicted only ~50% of wild NORTH individuals as obligate, whereas FULL features, which include the JHE signal, classified ~85% as obligate, consistent with the known phenotype distribution. This pattern indicates that the FULL feature set captures an additional layer of population‐level differentiation that is absent from the HIGH + LOW model.

Together, these results support two complementary genomic signals underlying diapause variation: a within‐population component of segregating variation (e.g., SLC25) that discriminates phenotypes locally, and a population‐level component of directional selection for obligate diapause (JHE) that differentiates northern from Central European populations. We note that FULL feature selection included all individuals, including NORTH, so the FULL feature set is not fully independent of the NORTH contrast, even though NORTH was excluded during LOPO training. Consequently, the HIGH + LOW model, which was developed without any NORTH individuals, provides the more conservative demonstration of within‐population predictive validity.

While our study was designed as a genomic discovery and prediction framework rather than a functional‐validation study, the candidate loci we identified represent high‐priority targets for downstream validation, foremost JHE (*IpsContig35*, locus 15284), given its convergent rGWAS‐ML support and established mechanistic role, together with the strongest within‐population predictor, the SLC25 locus (*LG03*, locus 8423). Fine‐mapping to narrow causal variants, allele‐specific expression assays by quantitative PCR, and direct juvenile‐hormone quantification by HPLC‐MS in facultative versus obligate individuals (Li et al. 2022; Ma et al. 2021; Ramirez et al. 2020) would strengthen the mechanistic interpretation and confirm the causative role of these loci.

The geographic distribution of facultative and obligate phenotypes reflects adaptation to local environmental conditions, particularly the avoidance of cold‐related mortality (Schebeck et al. 2017, 2022). Facultative diapausers dominate in permissive Central European habitats, where a longer growing season allows additional generations before photoperiod‐ and temperature‐cued diapause induction in late summer (Doležal and Sehnal 2007; Hofmann et al. 2024; Schebeck et al. 2022); because facultative diapause is governed primarily by photoperiod rather than temperature per se, the associations we detected are unlikely to reflect temperature‐driven plasticity, an interpretation reinforced by our controlled phenotyping. Consistent with this, roughly half of the wild HIGH and LOW individuals were predicted to express facultative diapause, whereas nearly all wild NORTH individuals (~97%) were predicted obligate, mirroring prior experiments (Schebeck et al. 2022). Obligate diapause is an effective strategy where short growing seasons make adult overwintering essential and pre‐imaginal stages suffer high cold mortality (Annila 1969; Koštál et al. 2011), producing the latitudinal voltinism cline observed in this species (Baier et al. 2007; Jönsson et al. 2011) and echoing adaptive differentiation across insects (Numata and Shintani 2025). The thermal contrast across our sites, milder, longer seasons in Central Europe versus extended winters at 64° N, is consistent with the near‐fixation of the JHE allele in NORTH and with the population‐level interpretation of that locus. Under continued climate warming, the proportion of facultative, potentially multivoltine individuals is expected to rise and spread into habitats previously dominated by obligate diapausers, with attendant increases in population density and outbreak risk.

Taken together, our findings highlight polygenic diapause regulation as a central ecological and evolutionary mechanism of seasonal synchronization, structured here into a within‐population segregating component and a population‐level, latitudinally selected component. The analytical pipeline we employed, integrating rGWAS with ML‐based feature selection and validated by permutation and cross‐population testing, provides a rapid and transferable tool for screening diapause phenotypes across the European range of *I. typographus*. Applied regularly, it can track shifts in phenotype ratios, assess voltinism potential, and provide early warning of multivoltine, outbreak‐prone populations (Hofmann, Kautz, and Schebeck 2025; Schebeck et al. 2022), with broad utility for forecasting insect responses to global change.

## Author Contributions

L.P.: conceptualization, methodology, software, validation, formal analysis, investigation, data curation, visualization, writing – original draft, writing – review and editing. E.J.D.: methodology, validation, writing – review and editing. K.N.‐B.: data curation, resources, writing – review and editing. A.S.: conceptualization, methodology. N.D.: conceptualization, methodology, investigation. C.S.: conceptualization, methodology, resources, review and editing, supervision. H.S.: conceptualization, methodology, resources, review and editing, supervision. G.J.R.: conceptualization, methodology, resources, writing – review and editing. M.S.: conceptualization, methodology, validation, formal analysis, investigation, resources, writing – review and editing, supervision.

## Funding

This work was supported by the Austrian Science Fund (FWF; grant P26749‐B25 to C.S.), the Autonomous Province of Bolzano‐South Tyrol (to H.S.) and the National Science Centre, Poland (NCN; grant 2018/30/E/NZ8/00105 to K.N.‐B.).

## Disclosure

Benefit‐Sharing: Benefits from this research are accumulated from the sharing of our data and results on public databases as described above.

## Ethics Statement

The authors have nothing to report.

## Conflicts of Interest

The authors declare no conflicts of interest.

## Supporting information


**Figure S1:** Analytical workflow overview.
**Figure S2:** BayPass IS model calibration via pseudo‐observed datasets (PODs). (A) Empirical Bayes Factor (BF) distribution for the FULL dataset (NORTH + HIGH + LOW; *n* = 296 *phenotyped* individuals, 2 phenotype populations: obligate and facultative). (B) Empirical BF distribution for the HIGH + LOW dataset (HIGH + LOW only; *n* = 239 *phenotyped* individuals), excluding NORTH where only obligate individuals were sampled. In both panels, the grey filled curve shows the null BF distribution derived from 10,000 SNPs simulated via PODs under the observed population covariance structure (omega matrix from the core BayPass model), representing the expected distribution under no phenotype association. Purple bars show the observed median BF across five independent IS model MCMC runs for each of the 11,867 SNPs. Dashed vertical lines indicate BF thresholds of 6 dB (moderate evidence, blue) and 10 dB (strong evidence, green). Percentages in the inset boxes indicate the empirical false positive rate, the proportion of null POD SNPs exceeding each threshold given the population structure and sample sizes of this dataset. SNP labels identify loci exceeding BF = 10 dB in each analysis.
**Figure S3:** Optuna hyperparameter tuning and optimization results.
**Figure S4:** Complete leave‐one‐population‐out (LOPO) cross‐validation results. Full per‐direction breakdown underlying Figure 6. (A, B) Cross‐population ROC within Central Europe for HIGH + LOW features (A; HIGH → LOW AUC = 0.78, LOW → HIGH AUC = 0.75) and FULL features (B; HIGH → LOW AUC = 0.74, LOW → HIGH AUC = 0.75); the diagonal denotes random. FULL features transfer within Central Europe comparably to HIGH + LOW SNP features, indicating that FULL features encode additional, rather than simply superior, information. (C, D) Predicted obligate proportion for NORTH phenotyped (C, *n* = 57) and NORTH wild (D, *n* = 93) individuals, by training population (Train HIGH, Train LOW, Train HIGH + LOW) and feature set (HIGH + LOW, green; FULL, magenta); error bars are bootstrapped 95% CIs and the dashed line marks the expected ~100% obligate. FULL features yield consistently higher NORTH obligate predictions than HIGH + LOW features across all training directions. With HIGH + LOW features, generalization to NORTH is partial and direction‐dependent (Train LOW: 79% phenotyped, 60% wild; Train HIGH: 37% and 34%), consistent with the low‐altitude population's segregating variation reaching somewhat further toward the northern phenotype than the high‐altitude population's.

**Table S1:** Final ipyrad statistics report.

**Table S2:** Complete 11k SNP‐set. Functional categories of annotated genes following the colour scheme of presented below.

## Data Availability

All preprocessing scripts and analytical related data and code are available in a public GitHub and ZENODO repositories (github.com/LucPalmieri/Diapause_genetic_markers, https://doi.org/10.5281/zenodo.17543185). De‐multiplexed raw sequences and related sample metadata are available at DDBJ Sequence Read Archive (DRA) under the Bioproject ID: PRJDB38037.
