# Supplementary material for: Genomic Predictors of Diapause Polymorphism in the Bark Beetle Ips typographus With Implications for Voltinism Potential and Outbreak Dynamics
Source: Mol Ecol. 2026 Aug 25;35(16):e70532. doi: 10.1111/mec.70532 (PMC13507623; doi:10.1111/mec.70532)
Supplement: Supplementary file 1 [file MEC-35-e70532-s002.docx]

**Supplementary material**

**Figure S1.** Analytical workflow overview.

**Figure S2.** BayPass IS model calibration via pseudo-observed datasets (PODs). (A) Empirical Bayes Factor (BF) distribution for the FULL dataset (NORTH + HIGH + LOW; n = 296 *phenotyped* individuals, 2 phenotype populations: obligate and facultative). (B) Empirical BF distribution for the HIGH+LOW dataset (HIGH + LOW only; n = 239 *phenotyped* individuals), excluding NORTH where only obligate individuals were sampled. In both panels, the grey filled curve shows the null BF distribution derived from 10,000 SNPs simulated via PODs under the observed population covariance structure (omega matrix from the core BayPass model), representing the expected distribution under no phenotype association. Purple bars show the observed median BF across five independent IS model MCMC runs for each of the 11,867 SNPs. Dashed vertical lines indicate BF thresholds of 6 dB (moderate evidence, blue) and 10 dB (strong evidence, green). Percentages in the inset boxes indicate the empirical false positive rate, the proportion of null POD SNPs exceeding each threshold given the population structure and sample sizes of this dataset. SNP labels identify loci exceeding BF = 10 dB in each analysis.

**Figure S4.** Complete leave-one-population-out (LOPO) cross-validation results. Full per-direction breakdown underlying Figure 6. (A, B) Cross-population ROC within Central Europe for HIGH+LOW features (A; HIGH→LOW AUC = 0.78, LOW→HIGH AUC = 0.75) and FULL features (B; HIGH→LOW AUC = 0.74, LOW→HIGH AUC = 0.75); the diagonal denotes random. FULL features transfer within Central Europe comparably to HIGH+LOW SNP features, indicating that FULL features encode additional, rather than simply superior, information. (C, D) Predicted obligate proportion for NORTH phenotyped (C, n = 57) and NORTH wild (D, n = 93) individuals, by training population (Train HIGH, Train LOW, Train HIGH+LOW) and feature set (HIGH+LOW, green; FULL, magenta); error bars are bootstrapped 95% CIs and the dashed line marks the expected ~100% obligate. FULL features yield consistently higher NORTH obligate predictions than HIGH+LOW features across all training directions. With HIGH+LOW features, generalization to NORTH is partial and direction-dependent (Train LOW: 79% phenotyped, 60% wild; Train HIGH: 37% and 34%), consistent with the low-altitude population's segregating variation reaching somewhat further toward the northern phenotype than the high-altitude population's.
